# Contemporary Echocardiographic Evaluation of Mitral Regurgitation and Guidance for Percutaneous Mitral Valve Repair

**DOI:** 10.3390/jcm12227121

**Published:** 2023-11-15

**Authors:** Davide Marchetti, Francesca Di Lenarda, Maria Laura Novembre, Pasquale Paolisso, Matteo Schillaci, Eleonora Melotti, Marco Doldi, Riccardo Terzi, Michele Gallazzi, Edoardo Conte, Valentina Volpato, Antonio Bartorelli, Daniele Andreini

**Affiliations:** 1 Division of University Cardiology, IRCCS Ospedale Galeazzi Sant’Ambrogio, 20100 Milan, Italy; davide.marchetti@grupposandonato.it (D.M.); eleonora.melotti@grupposandonato.it (E.M.); riccardo.terzi@unimi.it (R.T.); antonio.bartorelli@grupposandonato.it (A.B.); 2 Department of Clinical and Biomedical Sciences, University of Milan, 20100 Milan, Italy

**Keywords:** echocardiography, mitral regurgitation, mitraclip, transcatheter edge-to-edge repair

## Abstract

Mitral valve regurgitation (MR) is a multifaceted valvular heart disease. Echocardiography plays a central role in etiology assessment, severity quantification, treatment candidacy, outcome evaluation, and patient follow-up. In this review, we describe the comprehensive echocardiographic assessment of MR, including transthoracic (TTE) and transesophageal (TEE) approaches, 2D and 3D modalities, strain imaging, stress echocardiography, and artificial intelligence (AI) applications. Transcatheter edge-to-edge mitral valve repair (TEER) has been established as a key therapy for patients with severe, symptomatic MR and high surgical risk. TEER is performed under TEE guidance. We outline a practical overview of echocardiographic guidance on TEER.

## 1. Contemporary Echocardiographic Evaluation of Mitral Valve Regurgitation

To date, echocardiographic assessment of mitral valve regurgitation (MR) remains the primary diagnostic tool used to clarify the underlying pathophysiological mechanism, grade its severity, and describe consequent heart remodeling and prognosis [1]. Being a non-invasive, safe, and relatively cost-effective exam, it is an appropriate test to maintain a monitored follow-up.

### 1.1. MR Etiology Evaluation

Differential diagnosis between primary and secondary mitral regurgitation can be successfully carried out with two-dimensional echo (2DE). We refer to primary MR when the alteration to the valve anatomy concerns its leaflets: redundancy causing significant prolapse, Barlow disease, and leaflet flail following chordae rupture. Rheumatic mitral valve regurgitation has variable thickening of the leaflets, chordae fibrosis and consequent rigidity, and reduced motion of the posterior leaflet in diastole. Conversely, secondary or functional MR is caused by alterations or remodeling of the left ventricle or left atrium. Typically, left ventricle alterations which cause MR are either its dilatation, with consequent valvular tenting and leaflet malcoaptation, or segmental akinesia located in the posterior papillary muscle area which lead to tethering of the posterior leaflet. A key concept that has recently arisen is distinguishing proportionate versus disproportionate MR [2]. In fact, the COAPT [3] and MITRA-FR [4] trials have clearly shown that patients with severe regurgitation (EROA >0.3 cm^2^) and severely enlarged ventricles (proportionate MR phenotype) have worse response to medical and invasive treatments, while patients with severe regurgitation and non-severe LV dilatation (disproportionate MR phenotype) are reported to have better outcomes [5]. Annulus enlargement related to left ventricle dilatation and causing leaflet tethering corresponds to proportionate MR that can be reversed by anti-remodeling drug therapy and decrease in LV volume. Asymmetric ventricular wall contraction due to hypo- or akinesia following acute coronary syndrome causes a disproportionate MR instead, which has shown major benefit from procedural interventions and cardiac resynchronization.

Atrial MR is the result of atrial and consequent annulus dilatation in the presence of a normal left ventricle. Recently, Bursi et al. [6] have pointed out that atrial functional MR might also be the result of the interplay between ventricular and atrial disfunction, showing that the differentiation between atrial and ventricular functional MR could sometimes be simplistic.

Moreover, Huag et al. [7] have pointed out that secondary MR can induce leaflet alterations in terms of length and thickness following turbulence in intracardiac fluid dynamics, outlining a potential role of valve leaflet pathology in secondary MR as well.

Three-dimensional echo (3DE) allows the anatomical description of valve annulus and leaflets, contributing a comprehensive anatomical description of MR etiology: it can outline details regarding which segment of the mitral valve is interested by prolapse or flail; moreover, it can explain the distinction between organic MV lesions and other potential causes of MV regurgitation such as infective endocarditis or post-ischemic leaflet restriction. Three-dimensional echo allows precise annulus morphology and dimension assessment [8], which influences the choice of repairing technique, selection of annuloplasty ring, and liability of transcatheter mitral valve repair.

### 1.2. MR Severity Quantification

Basic quantification of mitral regurgitation includes qualitative, semi-quantitative, and quantitative parameters [9], which are summarized in Table 1.

#### 1.2.1. Qualitative Assessment

Qualitative assessment of mitral valve disease should start focusing on anatomy, as mentioned above. Abnormal mitral leaflet morphology includes thickening, calcification, redundancy, perforation, vegetations, other masses, and clefts. Abnormal sub-valvular morphology includes chordal rupture, thickening, fusion, vegetations, and masses, which should similarly be described in detail by size and location. Abnormal annular morphology includes dilation and calcification.

Once leaflet morphology has been described, leaflet motion should be investigated using the Carpentier classification system:

- Type I: normal leaflet motion, which may be seen in primary MR due to endocarditis, perforation, or clefts and in primary or secondary MR due to isolated annular dilation.
- Type II: excessive leaflet motion, which is most seen with MV prolapse or flail leaflet. Leaflet prolapse occurs when the leaflet body moves above the saddle-shaped annulus in systole, whereas leaflet flail occurs when a focal portion of the leaflet edge moves above the annulus and zone of coaptation. With flail leaflets, torn chords are usually visible.
- Type III: restricted leaflet motion, which is subclassified into restriction during both systole and diastole (IIIA) present in rheumatic MV disease, radiation- or drug-induced injury, or other inflammatory conditions. Restricted leaflet motion during systole only (IIIB) is present in functional MR.

First-sight evaluation takes into consideration the color Doppler jet’s intensity and area. Severe regurgitation usually corresponds to a wide jet with extension towards the atrial roof, to multiple jets, or to an eccentric, swirling jet that reaches the left atrium posterior wall (Coanda effect). However, color flow display depends on several hemodynamic factors, such as ventricular afterload, volume status, and co-existing valvopathies, which may be misleading in the process of quantifying the MR.

M-mode evaluation of the color Doppler jet enables temporal analysis, allowing better description of the timing and duration of an MR jet during systole. Functional MR usually shows early and late peaks with mid-systolic decrease and rheumatic MR usually presents end-systolic decrease in the flow, whilst a mitral valve prolapse regurgitation jet shows typical mid-late systole regurgitation.

#### 1.2.2. Semi-Quantitative and Quantitative Assessment

**Figure 1:**
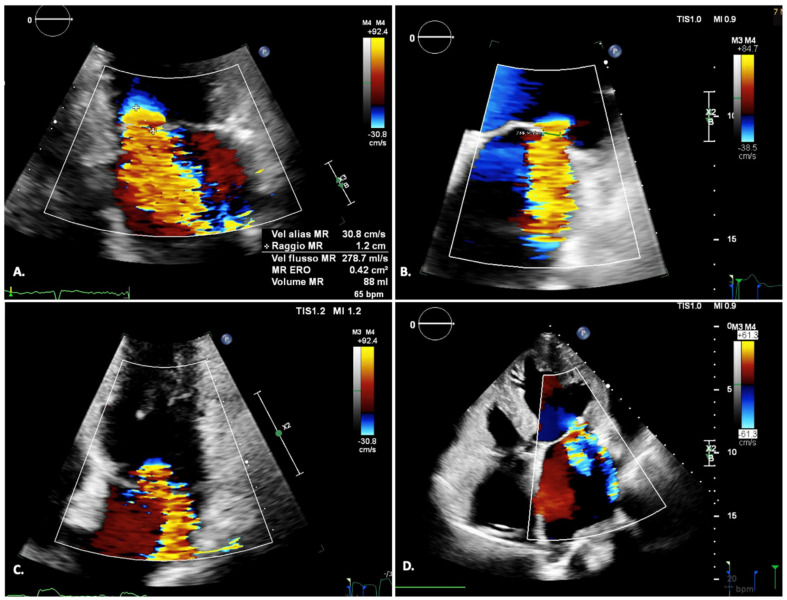
Echocardiographic severity parameters for mitral regurgitation; (**A**) Quantitative parameter: EROA; (**B**) Semi-quantitative parameter: Vena contracta; (**C**,**D**) Qualitative parameters: Color flow, Coanda effect.

Basic quantification of mitral regurgitation includes semi-quantitative and quantitative parameters [9] Figure 1.

Semi-quantitative parameters suggesting severe MR include: (1) vena contracta width above 7 mm, possibly measured in two perpendicular views; (2) pulsed Doppler mitral inflow E wave dominance (E/A > 1.5); (3) systolic flow reversal wave at pulsed-wave Doppler (PWD) on pulmonary veins; (4) mitral-to-aortic flow-velocity integral ratio > 1.4.

Quantitative parameters suggesting severe MR include: (1) effective regurgitant orifice area (EROA) bigger than 40 mm^2^. This cut-off value has been recently accepted by ESC [10], but in the case of functional MR it is underlined that geometrical assumption for EORA calculation can underestimate the severity of regurgitation in the case of elliptical orifice. In this specific setting, (1) EROA bigger than 30 mm^2^ can be regarded as severe regurgitation; (2) a regurgitation volume greater than 60 mL in primary MR and greater than 45 mL in secondary MR or in case of low flow condition; (3) a regurgitant fraction greater than 50% [11].

Pitfalls of the EORA method include errors in proximal isovelocity surface area (PISA) diameter measurement, especially in the case of a non-circular orifice. PISA may be influenced by adjacent structures (flow constraints) and it can change dimension during the systolic phase. Imprecision in PISA radius measurement is squared; thus, it can greatly influence the quantification of MR. Furthermore, MR quantification by EROA is not validated for multiple jets.

Other first-level supportive findings are atrial and ventricular chamber dilatation and elevated pulmonary systolic pressure; these are to be analyzed as possible insights into etiology.

**Figure 2:**
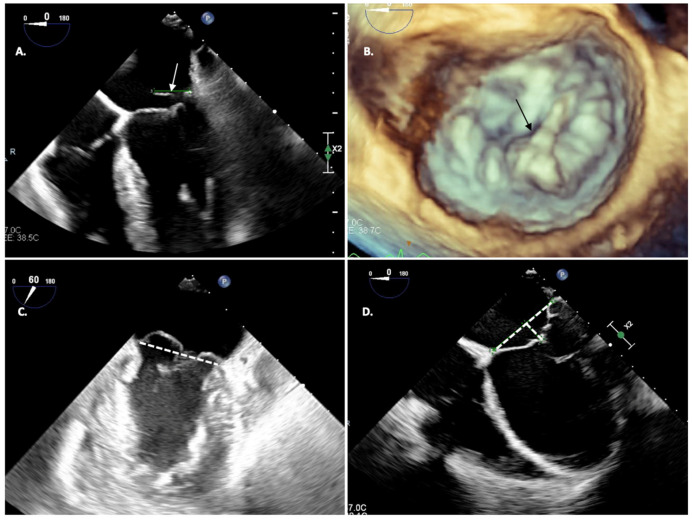
Echocardiographic evaluation of mitral regurgitation etiology; (**A**) PMV leaflet flail (white arrow); (**B**) PMV leaflet flail in 3D view (black arrow); (**C**) Bi-leaflet prolapse in Barlow disease (atrio-ventricular junction represented by white dashed line); (**D**) Tenting area in Secondary MR.

In acute MR, the left ventricle is not enlarged but the increased preload leads to high ventricular filling pressure and increased ejection fraction [12,13]. In chronic compensated patients, gradual LV and left atrium (LA) dilatation is observed while stroke volume is maintained. Decompensation comes with progressive reduction in EF [14], LV enlargement, and significant increase in LA pressure. Progressively, a rise in pulmonary arterial pressure may be observed, followed by right ventricle volume overload and tricuspid annular dilatation Figure 2.

In secondary MR, the underlying cause is LV and/or LA dysfunction or dilatation, which are more relevant than the degree of MR. Despite lower regurgitant volume, LA pressure is often elevated as opposed to primary MR [15].

As for 2DE, a high prevalence of inter-operator discordance in terms of grading is widely described in the literature [16], mostly in the presence of severe regurgitation. A comprehensive, multiparametric evaluation with a preference for quantitative parameters only has proven to increase concordance [11].

Three-dimensional echo has been demonstrated to improve precision in the definition of valvular defects by limiting geometric assumptions, especially when considering multiple and eccentric jets and non-circular regurgitant orifices. Three-dimensional vena contracta area (VCA) allows a better direct view of the jets and quantification of severity. Recently, Jungels et al. [17] have compared 2D PISA quantification and 3D VCA for functional MR in a prospective tertiary heart valve center registry, finding a significant correlation between the two (r = 0.93, *p* < 0.001) with an AUC of 0.98 (IC 0.97-1). They have specified a value of 3D VCA > 0.43 cm^2^ for a threshold of severe regurgitation.

The mitral valve apparatus is a complex structure and its morphology and shape vary through the cardiac cycle; thus, the regurgitation orifice can also show dynamic changes, especially in functional MR. Lozano-Edo et al. [18] have outlined that 3D VCA has a spatial–temporal modification over the cardiac systole that can challenge the assumption of a circular and fixed regurgitation orifice.

Although 3D VCA is a promising tool, robust data for quantification of severe MR in different setting are still lacking. For this reason, this evaluation should be interpreted with caution and correlated with other qualitative data.

### 1.3. Treatment Considerations

In case of severe MR, a comprehensive valve anatomy evaluation is crucial to candidate the patient to interventional treatments. Criteria in favor of cardiac surgery are the presence of normal left ventricular function and an in-range tele-systolic diameter (<40 mm) in symptomatic patients and reduced ejection fraction or dilated tele-systolic diameter (>45 mm) in asymptomatic patients [10].

When considering surgical mitral valve repair, several echocardiographic parameters can help to identify major failure risk [19]. In primary MR, unsuccessful repair has been reported in the presence of: (1) a large central regurgitant jet, (2) severe annulus dilatation (>50 mm), (3) involvement of more than three scallops, (4) extensive valve calcification. A lack of valve tissue predicts unsuccessful repair in rheumatic valve disease and after infective endocarditis with large leaflet perforation. In functional ischemic MR, the probability of recurrence of MR is major when: (1) the diastolic annulus diameter is larger than 37 mm, (2) there is a systolic tenting area of 1.6 cm^2^ or more, (3) there is a severe functional ischemic MR.

For the purpose of this review, we will later consider topics on percutaneous mitral valve repair, which have widened treatment availability for high-risk patients with excellent results and prognosis benefit [20].

### 1.4. Heart Remodeling Evaluation

The evaluation of heart remodeling caused by MR is a pivotal prognostic indicator. Three-dimensional assessment of ventricular and atrial volumes is extremely precise compared to cardiac magnetic resonance [21]. Semi-automated or automated 3D heart models are widespread among different vendors [22], allowing fast and reproducible volume quantification [23] and systolic function quantification.

Heart chamber deformation, which is defined as strain imaging, has been strongly linked to prognosis [24]. Left ventricular longitudinal strain (GLS), which describes left ventricle longitudinal deformation, has shown high sensitivity in evaluating borderline ventricular dysfunction [25,26] and its decrease below normal values usually precedes actual systolic dysfunction [27]; normal values are more negative than −18%, whereas values between −16% and −18% are considered borderline.

Peak left atrial longitudinal strain (PALS), also known as atrial reservoir, is an indicator of atrial compliance. PALS value reduction is linked to atrial dysfunction and is independently associated with all-cause mortality in patients with ventricular functional mitral regurgitation. This parameter is considered altered for values below 23% [28].

### 1.5. Stress Echocardiography and Functional MR

Stress echocardiography in apparently asymptomatic patients is helpful to confirm or exclude the presence of symptoms and to assess functional capacity.

When the degree of MR does not explain severe symptoms of the patient [29], exercise testing is important to highlight exercise-induced worsening MR or high pulmonary pressures and to observe the development of exercise-limiting symptoms. Moreover, it can disclose latent LV dysfunction and following limit in cardiac compensation resulting in an inadequate increase in ejection fraction and a larger end-systolic volume at exercise, therefore unmasking severe MR [30,31].

Finally, the absence of contractile reserve in asymptomatic patients with severe primary MR is linked to poor prognosis [32,33].

### 1.6. Artificial Intelligence and Machine Learning

Present and near-future perspectives involve artificial intelligence and machine learning [34]. Increased machine computing and database management have allowed the creation of algorithms that are processes or sets of rules to be followed in calculations/definitions/problem solving by a computer [35].

**Figure 3:**
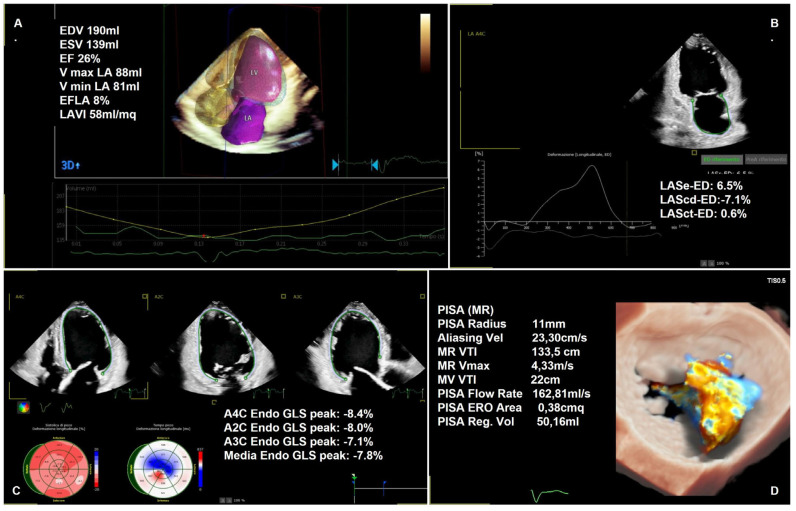
Advanced artificial intelligence-derived analysis; (**A**) Heart model—three-dimensional echcardiographic evaluation of left ventricular and left atrial volume and ejection fraction (i.e., dilated and reduced in function left ventricle); (**B**) PALS—peak atrial longitudinal strain analysis (i.e., reduced PALS: 6.5%); (**C**) GLS—global longitudinal strain (i.e., reduced GLS: −8.4%); (**D**) Automatic MR quantification via 3D analysis (i.e., severe MR).

In echocardiography, AI applications include standard section identification of cardiac anatomical structures, automatic recognition and segmentation of cardiac structures, and cardiac functional evaluation (e.g., 3D heart model) Figure 3.

Diagnosis via artificial intelligence is also under investigation in multiple pathological settings and, above all, in multiple mitral regurgitation [36] settings: (1) Automatic evaluation software for PISA can carry out an automated measurement of EROA and regurgitant volume; (2) Real-time 3D volume color-flow Doppler can quantify the regurgitation volume; (3) Valve morphology can undergo an automatic analysis by implementing automated measurements of the valve parameters, such as 2D area, commissural width, overlap width, anterior and posterior leaflet angle, non-planar angle, prolapse, valve height and volume, 3D leaflet area, 3D ring length, and height.

Finally, there is presently great interest in the use of unsupervised machine learning techniques to cluster patients on the basis of MR etiology, severity, and liability to treatment, enabling more standardized treatment protocols and more precise prognosis prediction [37].

Although those results are promising, expert clinical opinion continues to be crucial in treatment decision, clinical evaluation, and prognosis definition.

Table 2 summarizes different echo graphic techniques for MR evaluation.

**Table 1:** Main echocardiographic criteria for MR severity.

	Mild	Moderate		Severe
Qualitative				
Valve morphology	Mild leaflets alteration	Moderately abnormal leaflets		Severe prolapse, flail leaflet, ruptured papillary muscle, large perforation, severe retraction
Color flow jet	Small left atrium penetration	Moderate LA penetration/large late systolic		Deep LA penetration, Coanda effect
Flow convergence zone	Not visible/transient/small	Intermediate size and duration		Large throughout systole/flattened, hardly measurable in strongly eccentric jets
CW signal	Faint/partial, parabolic	Dense but partial (e.g., tele-systolic jet), parabolic and light density		Holosystolic, dense, triangular signal
MR duration (Color, M-mode)	Protosystolic or tele-systolic	Moderate and holosystolic or late systolic		Dense, holosystolic signal
Semi-quantitative				
Vena contracta width (mm)	<3	4–6		≥7
Pulmonary vein flow	Systolic dominance (diastolic dominance in AF and young patients)	Systolic blunting		Systolic flow reversal or Normal with low LA pressure
Mitral inflow	A-wave dominance	Variable		E-wave dominance (>1.5 cm/s)
Mitral TVI/Aortic TVI	<1.0	1.0–1.4		>1.4
Quantitative		Mild/ moderate	Moderate/ severe	
EROA (mm^2^)	<20	20–29	30–39	≥40 (≥30 in functional MR if elliptical orifice)
Regurgitant volume (mL)	<30	30–44	45–59	≥60
Regurgitant fraction (%)	<30	30–49		>50
Collateral findings				
LV and LA size (chronic severe MR)	Normal/mild LV dilation in secondary MR	Normal/mild dilation		Severe dilation
PA systolic pressure (mmHg)	Normal	Normal		Usually elevated

## 2. Echocardiographic Guidance for Percutaneous Mitral Valve Repair: A Practical Overview

Mitral regurgitation is the second most frequent valvular disease (VHD) in Europe.

In recent years, several opportunities for transcatheter mitral valve repair have been developed where surgery is at high risk or even contraindicated. Transcatheter edge-to-edge mitral valve repair (TEER) has now been established as a key therapy for severe symptomatic MR with high surgical risk [38].

Generally speaking, the indications should follow the same guidelines for surgical treatment of mitral regurgitation as recommended by ESC [10] and AHA/ACC [39].

Patients should be usually symptomatic with severe primary (or degenerative) MR with high surgical risk or severe secondary MR.

TEE guarantees the best view of the valve anatomy and can be integrated with 3D analysis [40]. Since the probe is as close as possible to the heart, TEE gives the best image quality both in 2D and 3D evaluation. It allows the visualization of leaflets, orifice, and sub-mitral apparatus, “en-face” views of the MV from an atrial and ventricular perspective, and calculation of fully sampled volume, limiting geometrical assumptions.

In addition, anatomical description via TEE includes the identification of which mitral scallops are involved in the defect’s mechanism. This detail proves useful in considering where to perform transcatheter edge-to-edge valve repair or surgery.

On the other hand, full evaluation of the ventricles and atria dimension and function is sometimes challenging; information on heart remodeling is limited and this is the reason why it should always follow the transthoracic evaluation.

Fusion imaging is a novel and still-overlooked technology that can acquire TEE and fluoroscopic images simultaneously, overlapping soft tissue imaging with fluoroscopic guidance for cardiovascular interventions. Fusion imaging can bring unquestionable advantages in the field of percutaneous TEER and experiences from selected tertiary heart valve centers have demonstrated favorable outcomes [41].

The last two decades have seen tremendous advances in the catheter-based treatment of structural heart diseases (SHD) thanks to: (1) the development of new percutaneous devices and the improvement of their performance; (2) increasing innovation and progress in imaging techniques that help guide these procedures. The development of a new subspecialty within cardiology, “the Interventional Imaging”, resulted in a new dedicated professional figure, the “SHD Interventional Imager”, with specific skills and competencies. The SHD Interventional Imager has become an integral part of the HEART TEAM by playing a central role in:

- Clinical decision-making regarding the appropriateness and feasibility of procedures;
- Pre-procedural planning;
- Intraprocedural guidance;
- Post-procedural follow-up.

### 2.1. Two-Dimensional TEE—Pre-Procedural Planning

Transthoracic echocardiography (TTE) is diagnostic in most cases, but transesophageal echocardiography (TEE) is always recommended when possible, especially if transthoracic ultrasound image quality is suboptimal.

**Figure 4:**
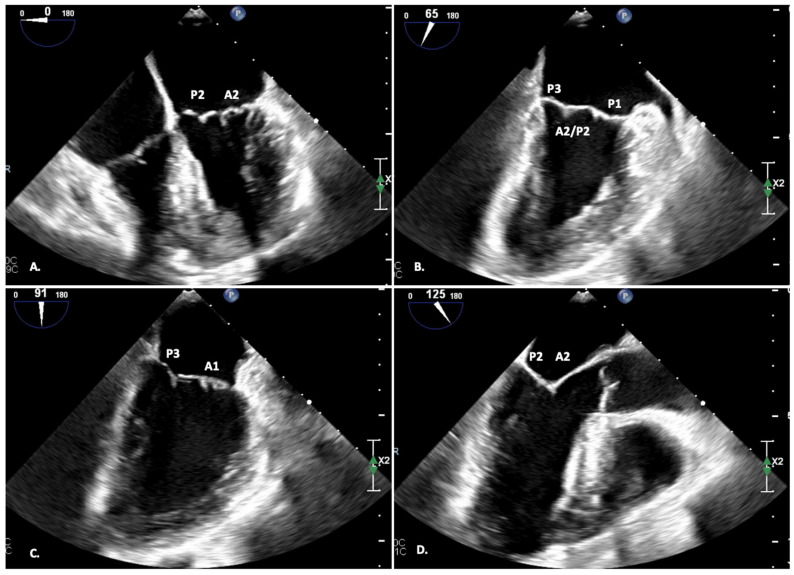
MV scallop definition during TEE analysis. Different views display the various scallops, better defining the valve’s defect. (**A**) Four-chamber view; (**B**) Commissural view; (**C**) Two-chamber view; (**D**) Three-chamber view.

The multiplanar 2D TEE allows visualization of the entire mitral valve apparatus with an analysis of the scallops Figure 4.

Depending on the TEE views, different scallops can be displayed:

- FOUR-CHAMBER view (0°): A2-P2;
- DUAL-CHAMBER view (90°): P1-A2-P3;
- BI-COMMISSURAL view (110°): A counterclockwise rotation of the probe displays A1-P1 (and the anterolateral commissure), while a clockwise rotation of the probe displays A3-P3 (and the posteromedial commissure);
- LVOT view (120°): A2-P2;
- TRANSGASTRIC view (0°): view of all the scallops; planimetric area of the MV.

In general, the anatomical criteria required [42] for the feasibility of the procedure are:

- Mitral valve area (≥4 cm^2^);
- Distance Fossa–Coaptation (≥4 cm);
- Flap length (≥10 mm), flap thickness (≤5 mm);
- Absence of calcification in the grasping area.

In the EVEREST [38,43] studies, different anatomical criteria were used for primary mitral regurgitation:

- Flail gap < 10 mm;
- Flail length < 15 mm.

Table 3 summarizes the main characteristics for TEER candidacy.

The COAPT [3] study used two anatomical inclusion criteria for secondary mitral regurgitation:

- Coaptation length ≥ 2 mm;
- Coaptation depth < 11 mm.

In general, central locations of mitral regurgitation (A2-P2) are easier to treat, whilst in the case of a defect located at the medial or lateral commissure, the procedure could be more difficult, because of the major presence of II type chordae and sub-valvular structures that lead to poor maneuverability of the system and entrapment [44].

In the EXPAND [45] registry, anatomical complexity was defined using the following criteria:

- Large coaptation gap (≥15 mm);
- Large flail gap (≥10 mm);
- Jets outside A2-P2;
- Small mitral valve area (MVA);
- Calcified landing zone;
- Minimal tissue of the flaps.

Patients meeting one or more of these criteria were less likely to achieve ≤1+ residual mitral regurgitation after edge-to-edge transcatheter repair.

However, these measures are sometimes difficult to evaluate using only conventional 2D images, presenting several limitations. In many cases of secondary mitral regurgitation, the flow convergence zone is not hemispherical and the regurgitant orifice has an oval or crescent shape. Therefore, the calculation derived from the PISA method using 2D echocardiography can easily underestimate the severity of regurgitation. To overcome these limitations, 3D TEE with multiplanar reconstruction provides a more accurate and reproducible assessment of these criteria.

### 2.2. Selection of TEER System—Pre-Procedural Planning

In Europe, there are currently two systems approved for percutaneous TEER: the MitraClip (Abbot) and Pascal (Edwards Lifesciences).

The MitraClip system was the first approved in the field both in Europe and America. At the present time, the fourth generation of the device is in use worldwide for organic and functional MR.

The MitraClip is composed of two rigid arms made of chromium–cobalt alloy with flexible “grippers” made of nitinol. Four different sizes of the Clip allow treatment for various anatomy and etiology of MR: NT and XT sizes are 4 mm wide and have 9 mm (NT) and 12 mm (XT) arm length; the recent releases NTW and XTW are 6 mm wide and have 9 mm (NTW) and 12 mm (XTW) length. The system is capable of continuous atrial pressure monitoring and has an independent leaflet capture mechanism for simultaneous or independent leaflet grasp.

The second-generation Pascal system is available at the present time for clinical use. It is composed of flexible nitinol arms and grippers. The original and wider size is P10, allowing up to 26 mm grasping length with 10 mm clasps and a central spacer that fills the regurgitant orifice. The Pascal ACE system, which has been approved since 2020, is smaller (6 mm length) but retains the same width of P10, allowing treatment of smaller or trickier anatomy. Pascal systems have continuous LA pressure monitoring and an independent leaflet capture mechanism.

With a broader-than-ever device availability, procedural planning of the treatment requires a deep interaction between the imaging cardiologist and interventional cardiologist to select the best TEER system for the right anatomy. A correct choice of device is crucial for the optimal result: while a bigger and wider system can grasp a larger surface of the leaflets, leading to more stable results, non-central regurgitation, smaller anatomies, or fibrotic leaflets need a smaller system to guarantee optimal treatment. The Clasp IID trial showed non-inferiority of the Pascal system vs. the MitraClip system for the treatment of severe degenerative MR [46].

### 2.3. TEE 3D—Pre-Procedural Planning

The 3D transesophageal echocardiogram can provide a comprehensive visualization of the different components of the mitral valve apparatus by overcoming the various weaknesses of a 2D multiplanar TEE.

Furthermore, this tool is particularly useful in the dialogue between interventional imager and surgeon, as it provides an “en-face” view seen from the perspective of the left atrium of the mitral valve that is identical to the surgical view in the operating room. This vision allows the user to, for example:

- Analyze the extent of commissural fusion in rheumatic MR or commissural prolapse in degenerative disease;
- Identify leaflets and scallops involved in degenerative myxomatous disease and diagnose the associated presence of chordal rupture or prolapse of several scallops;
- Establish the differential diagnosis between “indentation” and “cleft”;
- Reliably measure the mitral valve annulus, valve area, and tenting area

In addition, 3D color Doppler TEE and multiplanar reconstruction provide a direct measurement of regurgitant orifice area and the jet direction, which can improve the accuracy of regurgitant classification.

## 3. Procedural Phase

The procedure is performed under general anesthesia and under TEE guidance with a 2D/3D probe.

**Figure 5:**
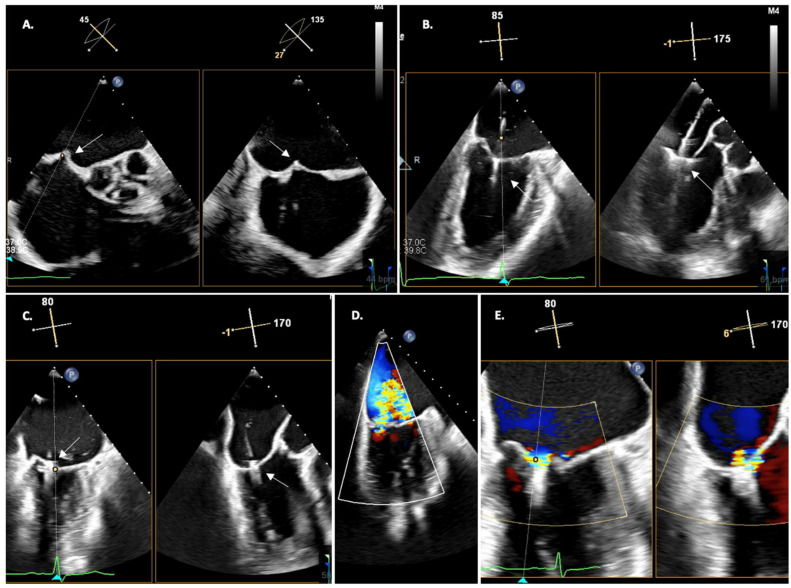
Echocardiographic procedural views of MitraClip implantation; (**A**) Trans-septal puncture (pre-puncture evaluation, white arrow); (**B**) Clip positioning (open clip pointed by white arrow); (**C**) Grasping (closed clip pointed by white arrow); (**D**) Pre-clip severe MR color flow; (**E**) Post-clip mild residual regurgitation.

Unlike surgery, where TEE is only involved in the pre-surgical assessment phase and outcome assessment, TEE is mandatory to guide the procedure of TEER [47,48] Figure 5.

### 3.1. Trans-Septal Puncture

Determining the optimal site for trans-septal puncture is a crucial initial role of the 3D TEE guide for the MitraClip implantation as it fixes the position of the steerable guiding catheter and affects the mobility of the clip delivery system.

The key TEE views of the septum are:

- The BICAVAL view (110°) for superior-inferior axis;
- The SHORT-AXIS view (45°) of the aortic valve for antero-posterior axis;
- The FOUR-CHAMBER view for puncture height.

In general, the puncture should be located posteriorly and superiorly in the fossa ovalis.

In patients with primary MR, the puncture site height should be 4–5 cm from the mitral annulus. Conversely, in patients with secondary MR due to flap tethering, the height may be reduced because the flap coaptation plane shifts toward the left ventricle.

However, a height from flap coaptation of <3.5 cm should be avoided because it may make the procedure difficult.

1. BICAVAL view: The puncture needle within the sheath is advanced from the right femoral vein down to the superior vena cava (SVC). Under TEE and fluoroscopic guidance, the puncture needle is retracted along the intra-atrial septum, down the SVC to the junction of the muscular and membranous septum.
2. SHORT AXIS view: The trans-septal sheath and needle assembly should be rotated clockwise until the tenting is visible in the posterior part of the membranous septum. Because the mitral valve coaptation line is in the posterior LA, a posterior puncture allows the system to maneuver almost only laterally and medially, without excessive antero-posterior movement, as the system is advanced or retracted. If the puncture is too anterior, the guidewire may be too close to the aorta, resulting in an “aortic hugger” scenario where the system runs nearly parallel to the aorta in the LVOT view.
3. FOUR-CHAMBER view: In this view, it is possible to measure the ideal distance between the puncture point in the septal wall and the mitral annular plane. An excessively high puncture could create too much distance to the MV coaptation line; the height of the system could be too short to reach and grip the flaps. If punctured too low, there could be inadequate space to orient the system and to grasp the leaflets, as the catheter tip may be just above and too close to the plane of the mitral annulus.
4. The guide catheter is then inserted into the left atrium. The disappearance of the tenting on the inter-atrial septum is an indicator of complete crossing; the dilator can be removed when the guide catheter crosses the inter-atrial septum by at least 2 cm. The tip of the sheath and dilator should be clearly visualized on TEE, which allows for safe advancement.

Echo monitoring is also fundamental during this procedure for early identification of complications such as atrial roof perforation, thrombus formation, or ST-segment elevation due to air embolism in the right coronary artery.

Fusion imaging can provide added value during trans-septal puncture [49]:

- The catheter and fossa ovalis are displayed simultaneously on the fluoroscopic screen and the transesophageal echocardiogram, allowing operators to understand the spatial position of the catheter in superior-inferior and anterior-posterior directions.
- The downward pull of the catheter in the direction of the inter-atrial septum can be monitored using the 2D bicaval view superimposed on the left anterior oblique (LAO) view, in which the lateral profile of the septum is well visualized.

### 3.2. Clip Implantation

For the spatial orientation of the system in relation to the mitral valve, the following TE views are used [50]:

- BI-COMMISSURAL view (60°) for information on the medial/lateral orientation;
- LVOT view (120°) for anterior/posterior orientation of the clip within the mitral orifice;
- THREE-DIMENSIONAL EN-FACE-view of the mitral valve from the atrial side, with or without color Doppler, to understand the origin and direction of regurgitant jet/jets.

**Figure 6:**
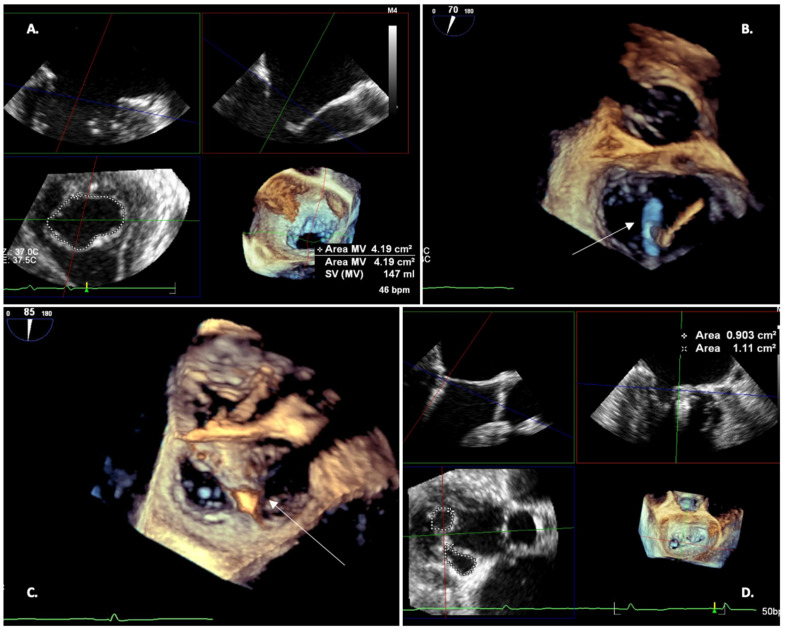
Three-dimensional TEE during MitraClip implantation; (**A**) Pre-procedural MV area evaluation; (**B**) Three-dimensional view of clip positioning (open clip pointed by white arrow); (**C**) Three-dimensional view of grasping (closed clip pointed by white arrow); (**D**) Post-procedural residual MV area evaluation.

Percutaneous MV repair is carried out following the steps described below:

1. Following atrial septal puncture, the delivery system is advanced into the LA.
2. The device tip should be advanced under echocardiographic guidance to avoid contact with the LA wall. Since the greatest distance from the septal puncture to the lateral wall of the LA in many patients is in the direction of the left superior pulmonary vein, the clip should be advanced in this direction, parallel to the mitral valvular plan.
3. The clip should be placed in the LA, over the center of the mitral valve, pointing toward the apex of the LV in the BI-COMMISSURAL view and above the coaptation line in the LVOT view. A 120–140° X-Plane view allows simultaneous projection. The trajectory must ensure perpendicularity to the plane of the mitral valve (MV). Once proper axial alignment is achieved on the target lesion, the device arms are unfolded and oriented perpendicular to the coaptation line. Off-axis grasping of the leaflets can lead to valve distortion which can lead to worsening mitral regurgitation or leaflet injury. The LVOT view should show both clip arms of the same length if the clip arm orientation is perpendicular to the coaptation line. The BI-COMMISSURAL view should show the clip as a bar, as this plane is oriented 90 degrees to the LVOT view and perpendicular to the clip arms.
4. The clip should be oriented perpendicularly to the coaptation line; a 3D EN-FACE view helps to orientate when viewed from the LA. Clockwise rotation of the system causes clockwise rotation of the clip arms; conversely, when viewed from the LV, counterclockwise rotation of the system results in clockwise rotation of the clip arms.
5. The system is then advanced through the MV with the clip arms partially open. Once below the MV plane, the arms are fully opened.
6. The clip should be oriented to achieve perpendicularity to the coaptation line of the flaps using the 3D EN-FACE view.
7. Leaflet grasping: After partial closure of the clip, the delivery catheter is retracted to grab the leaflets. The entire clip is then closed to about 60 degrees.
8. Correct flap insertion and clip location at the origin of the MR jet should be ensured. The LVOT/4CH view can confirm that mitral leaflets are adequately captured, whilst the BICOMMUSSUAL view is used to ensure that the flap on each side bisects the clip. Finally, an EN-FACE 3-D view shows the presence of a stable double orifice and the reduction of the MR jet.
9. After adequate insertion of the flaps at 60 degrees of clip closure has been confirmed, the clip should be slowly closed further until the flaps are covered. The degree of MR reduction should be evaluated with color Doppler in all TEE views. Once the result is satisfactory, the device is detached from the delivery system Figure 6.

During clip implantation, continuous monitoring is essential for early recognition of possible complications such as intracardiac thrombosis on devices, cardiac tamponade, worsening of MR due to damage to flap or cord, loss of flap insertion or clip detachment, and iatrogenic mitral stenosis.

### 3.3. Post-Procedure

An optimal outcome should ensure a good balance between adequate MR reduction, without distortion of the valve, and the risk of developing iatrogenic stenosis. MV area ≤ 1.5 cm^2^ and a trans-mitral pressure gradient >5 mmHg indicate significant mitral stenosis.

Quantification of residual MR after Clip implantation has proven challenging using 2D TEE [51]. The PISA method, commonly used to evaluate native MR, is not feasible for residual MR after Clip implantation, because the residual jet can have multiple orifices and eccentric jets. In this case, 3D regurgitant orifice area may be a feasible and reliable method for the quantification of residual MR after Clip implantation [52]. PW pulmonary venous flow assessment can provide useful information to indirectly determine the severity of residual MR.

After the end of the procedure, it is suggested to control for clip stability and cardiac tamponade and to assess the residual shunt and size of the iatrogenic atrial septal defect (ASD), which is usually not clinically significant after retrieval of the delivery system.

## 4. Conclusions

Imaging plays a central role in all the steps of structural heart disease from the clinical decision-making before the procedure to deciding on appropriateness and feasibility to the intraprocedural guidance and post-procedural follow-up.

Echocardiographic assessment of mitral regurgitation is a complex world where “traditional” and well-proven evaluation meets an ever-evolving and exciting world of new technologies such as latest-generation three-dimensional echo hardware and software, artificial intelligence applications, and fusion imaging.

A contemporary evaluation of mitral regurgitation must consider multiple qualitative, semi-quantitative, and quantitative parameters and should embrace new tools that will soon be regarded as benchmarks, such as automated strain imaging, automated 3D volumes, and vena contracta 3D.

Deep interconnection between imaging cardiologists and interventional cardiologists is crucial for the treatment of mitral valve regurgitation. The synergy of these two players is the basis for transcatheter edge-to-edge mitral valve repair, as the procedure is possible only with excellent step-by-step echocardiographic guidance.

## Figures and Tables

**Table 2:** Role of different echocardiographic approach in MR evaluation.

Settings			
	Primary MR	Secondary MR	Interventional Echo
2D transthoracic	MR quantification		-
	Acute vs. chronic MR		
	Etiology: prolapse (AML-PML)/flail/rheumatic disease/degenerative disease	Etiology: ischemic vs. non ischemic (atrial MR/ventricular MR	
	Associated valve/heart disease		
	LV/LA function		
	Hemodynamic consequences		
3D transthoracic	MR quantification (especially eccentric jets)		-
	Better anatomical description		
2D/3D transesophageal	MR quantification and confirmation of severity (eccentric jets/poor transthoracic window)		- Guidance in edge-to-edge repair - Guidance in artificial chord implantation - Guidance in TMVR (transcatheter mitral valve replacement) - Guidance during surgery to access post-procedural residual defect - Guidance in annuloplasty
	Etiology: Exclusion of thrombi/infective endocarditis; flail gap and width; leaflet-to-annulus index; calcifications	Etiology: coaptation depth/length; annular dimensions	
	Exclusion of contraindications for planned procedure		
3D transesophageal	Determination of morphological suitability for a specific transcatheter procedure		
	Better view of valve’s anatomy and possibility of obtaining ‘en-face’ surgical view		
Stress echo	Confirmation/exclusion absence of symptoms during exercise		-
	Latent MR disclosure		
	Prognosis assessment analyzing contractile reserve		
New automated analysis	Peak atrial longitudinal strain for early assessment of atrial dysfunction		-
	GLS as early assessment of ventricular damage		
	Heart volumes for prognosis		

**Table 3:** Summary of criteria for TEER candidacy.

	Optimal	Reasonable	Inappropriate
Lesion location	Central (A2-P2 scallop)	Medial (A3-P3) Lateral (A1-P1)	Commissure, Cleft, Perforation, Vegetation
Lesion Extension Primary MR: Flail Width Flail Gap Secondary MR Coapt. Length Coapt. Depth	<15 mm <10 mm <11 mm <3 mm	>15 >11 mm 1–3 mm	Complex Barlow disease No coaptation
Calcification	None	Not involving grasping area	Extensive
MV Area	>4 cm^2^	>3 cm^2^ if good leaflet mobility and normal mean gradient	<3 cm^2^ or MG > 5 mmHg
Posterior leaflet length	>10 mm	7–10 mm	<7 mm
Leaflet mobility	Normal	Systolic restriction (Carpentier IIIb)	Systo-diastolic restriction (Carpentier IIIa)
Leaflet Thickness	Normal	<5 mm	>5 mm

## Data Availability

Not applicable.

## Abbreviations

MR	mitral regurgitation
TTE	transthoracic echocardiography
TEE	transesophageal echocardiography
AI	artificial intelligence
TEER	transcatheter edge-to-edge mitral valve repair
PWD	pulsed-wave doppler
EROA	effective regurgitant orifice area
LVEF	left ventricle ejection fraction
LA	left atrium
2DE	two-dimensional echo
3DE	three-dimensional echo
GLS	left ventricular longitudinal strain
PALS	peak left atrial longitudinal strain
PISA	proximal isovelocity surface area
ESC	European Society of Cardiology
SHD	Structural Heart Disease
AHA/ACC	American Heart Association/American College of Cardiology
